# Major Histocompatibility Complex Class II (DRB3) Genetic Diversity in Spanish Morucha and Colombian Normande Cattle Compared to Taurine and Zebu Populations

**DOI:** 10.3389/fgene.2019.01293

**Published:** 2020-01-10

**Authors:** Michel David Bohórquez, Diego Ordoñez, Carlos Fernando Suárez, Belén Vicente, Carmen Vieira, Julio López-Abán, Antonio Muro, Iván Ordóñez, Manuel Alfonso Patarroyo

**Affiliations:** ^1^Microbiology Postgraduate Programme, Universidad Nacional de Colombia, Bogotá, Colombia; ^2^Molecular Biology and Immunology Department, Fundación Instituto de Inmunología de Colombia (FIDIC), Bogotá, Colombia; ^3^PhD Programme in Biomedical and Biological Sciences, Universidad del Rosario, Bogotá, Colombia; ^4^Faculty of Agricultural Sciences, Universidad de Ciencias Aplicadas y Ambientales (UDCA), Bogotá, Colombia; ^5^Basic Sciences Department, School of Medicine and Health Sciences, Universidad del Rosario, Bogotá, Colombia; ^6^Infectious and Tropical Diseases Research Group (e-INTRO), Biomedical Research Institute of Salamanca-Research Centre for Tropical Diseases at the University of Salamanca (IBSAL-CIETUS), Faculty of Pharmacy, University of Salamanca, Salamanca, Spain

**Keywords:** MHC, *BoLA-DRB3*, genetic diversity, peptide-binding region, genetic resistance, cattle

## Abstract

Bovine leukocyte antigens (BoLA) have been used as disease markers and immunological traits in cattle due to their primary role in pathogen recognition by the immune system. A higher MHC allele diversity in a population will allow presenting a broader peptide repertoire. However, loss of overall diversity due to domestication process can decrease a population’s peptide repertoire. Within the context of zebu and taurine cattle populations, *BoLA-DRB3* genetic diversity in Spanish Morucha and Colombian Normande cattle was analyzed and an approach to estimate functional diversity was performed. Sequence-based typing was used for identifying 29, 23, 27, and 28 alleles in Spanish Morucha, Nariño-, Boyacá-, and Cundinamarca-Normande cattle, respectively. These breeds had remarkably low heterozygosity levels and the Hardy-Weinberg principle revealed significant heterozygote deficiency. *F_ST_* and *D_A_* genetic distance showed that Colombian Normande populations had greater variability than other phenotypically homogeneous breeds, such as Holstein. It was also found that Spanish Morucha cattle were strongly differentiated from other cattle breeds. Spanish Morucha had greater divergence in the peptide-binding region regarding other cattle breeds. However, peptide-binding region covariation indicated that the potential peptide repertoire seemed equivalent among cattle breeds. Despite the genetic divergence observed, the extent of the potential peptide repertoire in the cattle populations studied appears to be similar and thus their pathogen recognition potential should be equivalent, suggesting that functional diversity might persist in the face of bottlenecks imposed by domestication and breeding.

## Introduction

The major histocompatibility complex (MHC) is a primary component of the adaptive immune system that offers a unique alternative for addressing both immunological and evolutionary biological issues. The MHC consists of a group of loci in jawed vertebrates encoding molecules which are fundamental for regulating the immune response (Klein et al., 2007). Class I and class II genes comprise one subset of these loci encoding the cell surface glycoproteins necessary for T-lymphocytes to recognize antigenic peptides on cell surfaces (Rock et al., 2016). While class I molecules are expressed by all nucleated cells and present peptides from intracellular proteins to CD8^+^ T-cells, class II molecules are expressed by professional antigen-presenting cells and present peptides derived from extracellular proteins to CD4^+^ T-cells, thereby playing a key role in defence against pathogens (Klein et al., 2007; Neefjes et al., 2011).

The MHC in cattle (also known as bovine leukocyte antigen-BoLA) is located on chromosome 23 and has the overall structure characterizing other mammals’ MHC (Takeshima and Aida, 2006). DR and DQ genes encode the molecules within the BoLA class II region that bind the peptides which will be presented to T-lymphocytes, representing the main class II restriction elements for CD4^+^ T-cells. The BoLA-DQ region comprises DQA and DQB loci, which may vary in number depending on the haplotype; this enables additional diversity by means of intrahaplotype and interhaplotype pairing of DQA and DQB molecules (Andersson and Rask, 1988; Glass et al., 2000; Norimine and Brown, 2005). BoLA-DR consists of the monomorphic *BoLA-DRA* locus and three DRB loci, of which the *BoLA-DRB3* gene is the only one known to be fully functional (Burke et al., 1991).

*BoLA-DRB3* is the most polymorphic bovine MHC gene; 136 different alleles have been reported to date (Maccari et al., 2017). Such polymorphisms are mainly located in the β1 domain’s peptide-binding region (PBR), which is encoded by exon 2 and has been used for defining *BoLA-DRB3* alleles (Sigurdardottir et al., 1991). Because the amino acids (aa) forming the PBR determine MHC-presented peptides’ binding affinity, different alleles will bind a different repertoire of peptides, thus influencing immune response variability (Baxter et al., 2009; van Deutekom and Kesmir, 2015). Similarities between *BoLA-DRB3* PBR are used as criteria for developing in silico pan-specific methods for predicting MHC–peptide-binding affinity (Nielsen et al., 2008; Hoof et al., 2009). *BoLA-DRB3* divergence can be used for estimating the size of peptide-binding repertoire. Thus, individuals or populations having highly divergent *BoLA-DRB3* alleles will have a broader peptide-binding repertoire than those having very similar alleles (Klein et al., 2007; Lenz et al., 2013). Furthermore, peptide-binding differences among populations could be estimated by correlating variation patterns among their *BoLA-DRB3* allele repertoires.

Ascertaining cattle’s *BoLA-DRB3* allele frequency distribution in different regions worldwide has been used for executing infectious disease control programmes (Maillard et al., 2003) and can be applied to developing vaccines having a wider range of protection (Patarroyo et al., 2011). Different *BoLA-DRB3* alleles have been associated with variations in susceptibility to infectious diseases (Dietz et al., 1997; Acosta-Rodriguez et al., 2005; Martinez et al., 2006; Nascimento et al., 2006; Kulberg et al., 2007; Juliarena et al., 2008; Nikbakht et al., 2016; Carignano et al., 2017), vaccine responses (Garcia-Briones et al., 2000; Rupp et al., 2007; Baxter et al., 2009; Gowane et al., 2013) and production traits (Sharif et al., 1999). *BoLA-DRB3* genetic diversity has been characterized in both widespread and autochthonous creole cattle breeds (Takeshima et al., 2001; Takeshima et al., 2002; Takeshima et al., 2003; Baxter et al., 2008; Wang et al., 2008; Giovambattista et al., 2013; Takeshima et al., 2014; Takeshima et al., 2015a).

MHC diversity is of great interest for breeders, population geneticists and evolutionary biologists, considering that different degrees and MHC variability patterns reflect evolutionary processes such as adaptation, selection (natural, sexual or artificial) and drift within and between populations (Goszczynski et al., 2014; Takeshima et al., 2014). Several studies have shown that decreased MHC variability might be caused by population bottlenecks (Bollmer et al., 2007; Bollmer et al., 2011; Mason et al., 2011; Zhang et al., 2016). On the contrary, a high level of diversity could be maintained by balancing selection driven by pathogens or other mechanisms due to MHC function regarding pathogen recognition, despite extreme population bottlenecks (Edwards and Hedrick, 1998; Garrigan and Hedrick, 2001; Aguilar et al., 2004; Borg et al., 2011; Moutou et al., 2013; Newhouse and Balakrishnan, 2015). Although functional polymorphism may be narrower than genetic polymorphism in pigs (Moutou et al., 2013), most studies on domestic animals’ MHC have focused on genetic polymorphism; however, it remains unknown whether bottlenecks associated with domestication and breeding could have reduced MHC functional diversity.

Despite advances in characterizing *BoLA-DRB3* genetic diversity in cattle, information about allele frequency is not available for some breeds (only for a few out of over 800 breeds of cattle recognized worldwide *via* sequence-based typing) (Takeshima et al., 2018). The few MHC alleles in domestic animals contrasts with those of other species (Nino-Vasquez et al., 2000; Suarez et al., 2006; Lopez et al., 2014; Maccari et al., 2017; Shiina et al., 2017). However, a smaller amount of alleles does not necessarily mean reduced functionality, in fact two species can have very similar functional repertoire size despite varying regarding the amount of alleles (Suarez et al., 2017).

This study has thus been aimed at determining whether domestic cattle breeding has decreased actual MHC variability. MHC diversity of two new breeds has been characterized. The Normande breed was chosen out of convenience. Spanish Morucha, a breed having relatively low human intervention, has been used here as an approach to what could be considered a natural cattle population. *BoLA-DRB3* genetic diversity, structure, differentiation, and selective pressure in Colombian Normande and Spanish Morucha breeds was characterized and compared to worldwide taurine and zebu breeds. Each cattle breed’s *BoLA-DRB3* PBR variability and covariation was then analyzed as an approach to compare potential peptide-binding repertoire size and indication of functional diversity. Genetic divergence patterns based on population genetics and PBR variability analysis were similar to that observed for functional divergence; however, the potential peptide-binding repertoire was very similar for all breeds. These results provide an insight into MHC evolution and contribute toward efforts at describing bovine MHC diversity according to breed and location.

## Materials and Methods

### Study Population and DNA Extraction

Whole blood was collected aseptically from the coccygeal vein of 165 Normande and Morucha cattle. Considering that Colombian and Spanish cattle farming is characterized by extensive livestock production systems having a relatively few animals per herd, phenotypically purebred animals were sampled from the regions having the greatest amount of purebred cattle in Colombia (Normande) and Spain (Morucha), sampling the most representative amount of farms from each region (**Table 1**). The herds and purebred animals analyzed were sampled randomly, avoiding related individuals/farms for each region. Normande cattle samples came from Colombia’s Nariño (n = 30), Cundinamarca (n = 41), and Boyacá departments (n = 40). Morucha cattle samples (n = 54) came from Spain’s Salamanca province. A PureLink Genomic DNA Mini Kit (Invitrogen, Carlsbad, CA, USA) was used for extracting genomic DNA (gDNA), following the manufacturer’s instructions. Previously reported data regarding further 17 taurine and zebu populations from Asia and South America was also included for comparison (Takeshima et al., 2003; Giovambattista et al., 2013; Takeshima et al., 2015a; Takeshima et al., 2015b; Takeshima et al., 2018). **Table 1** gives general information about the populations analyzed. The Universidad de Ciencias Aplicadas y Ambientales (UDCA, Bogotá) Research Ethics Committee (Minute No. 201901) approved this study.

**Table 1:** General information regarding the 21 cattle populations analyzed in this study.

Breed	Acronym	N	Amount of farms	Sampling country	Type	Source
Normande	Cn_Nor	30	6	Colombia (Nariño)	taurine	This study
Normande	Cc_Nor	41	5	Colombia (Cundinamarca)	taurine	This study
Normande	Cb_Nor	40	3	Colombia (Boyacá)	taurine	This study
Morucha	Sp_Mor	54	15	Spain	taurine	This study
Nellore	Bo_Ne	116	2	Bolivia	zebuine	(Takeshima et al., 2018)
Gir	Bo_Gir	107	2	Bolivia	zebuine	(Takeshima et al., 2018)
Nellore x Brahman	Pe_Ne-Br	195	1	Peru	zebuine	(Takeshima et al., 2018)
Holstein	Ar_Ho	424	4	Argentina	taurine	(Takeshima et al., 2015b)
Holstein	Bo_Ho	159	2	Bolivia	taurine	(Takeshima et al., 2015b)
Holstein	Pa_Ho	127	5	Paraguay	taurine	(Takeshima et al., 2015b)
Holstein	Pe_Ho	133	2	Peru	taurine	(Takeshima et al., 2015b)
Holstein	Ch_Ho	113	5	Chile	taurine	(Takeshima et al., 2015b)
Hereford	Ch_He	49	2	Chile	taurine	(Takeshima et al., 2015a)
Hereford x Jersey	Ch_He-Je	65	1	Chile	taurine	(Takeshima et al., 2015a)
Wagyu	Ch_Wa	81	2	Chile	taurine	(Takeshima et al., 2015a)
Yacumeño	Bo_ Yac	113	4	Bolivia	taurine	(Giovambattista et al., 2013)
Hartón del Valle	C_HdV	66	1	Colombia	taurine	(Giovambattista et al., 2013)
Holstein	Ja_Ho	101	Random collection	Japan	taurine	(Takeshima et al., 2003)
Shorthorn	Ja_Sh	100	Random collection	Japan	taurine	(Takeshima et al., 2003)
Jersey	Ja_Je	69	Random collection	Japan	taurine	(Takeshima et al., 2003)
Black	Ja_Bl	201	Random collection	Japan	taurine	(Takeshima et al., 2003)

### Polymerase Chain Reaction, Cloning and Sequencing

Sequence-based typing was used for identifying *BoLA-DRB3* alleles. The *BoLA-DRB3* gene’s exon 2 was amplified by polymerase chain reaction (PCR), using a high-fidelity polymerase along with DRB3F and DRB3R primers (Ledwidge et al., 2001). The PCR reaction mixture contained 1X *Pfx* amplification buffer, 300 μM of each dNTP, 0.45 μM of each primer, 1 mM MgSO_4_, 1 U Platinum *Pfx* DNA Polymerase (Invitrogen), and 50 ng gDNA in a 50 μl final volume. Two independent reactions were performed for each sample, following Lenz and Becker’s recommendations (Lenz and Becker, 2008) to avoid the formation of chimeric products; the thermal profile was as follows: a denaturation step at 94°C for 5 min followed by 30 cycles of 94°C for 30 s, 64°C for 30 s, and 68°C for 1 min. The Wizard SV Gel and PCR Clean-Up System (Promega, Madison, WI, USA) was used for purifying PCR products which were then sequenced using a BigDye Terminator kit (Macrogen, Seoul, South Korea and Sequencing Service of the University of Salamanca, Spain) in both directions (using the DRB3F and DRB3R primers).

Another two PCR products were purified and ligated independently into pELMO vector for those animals to which a genotype could not be assigned from the PCR products sequences (Ramos et al., 2017). The ligated PCR products were used for transforming *Escherichia coli* TOP10 chemically competent cells (Invitrogen); colony PCR, with the DRB3F and DRB3R primers, was used for screening the resulting colonies for positive recombinant clones. The colony PCR reaction mixture contained 1X NH_4_ reaction buffer, 250 μM of each dNTP, 0.25 μM of each primer, 1.5 mM MgCl_2_, 1 U BIOLASE DNA polymerase (Bioline, London, UK) in a final 10 μl volume. A Zyppy Plasmid Miniprep Kit (Zymo Research Corporation, Irvine, CA, USA) was used for plasmid extraction, following the manufacturer’s instructions. A BigDye Terminator kit (Macrogen) was used for sequencing clones in both directions, using ccdBsec-F and ccdBsec-R external primers (Ramos et al., 2017). At least eight clones were sequenced for each animal.

### Sequence Analysis

CLC Main Workbench v.3.6.5 software (CLC bio, Aarhus, Denmark) was used for assembling each independent PCR and clone sequences (manually edited if necessary) and heterozygous positions were identified for producing a consensus containing IUPAC ambiguity codes. The genotype for each animal was assigned by comparing these sequences with *BoLA-DRB3* allele sequences reported in the IPD-MHC database (Maccari et al., 2017) using HAPLOFINDER (Miltiadou et al., 2003), according to Baxter et al., 2008.

### Genetic Diversity Measurements, Hardy-Weinberg Equilibrium and Selection

Allele frequencies and the amount of alleles (N_a_) were obtained by direct counting; 95% confidence intervals (95%CI) were calculated for allele frequencies, according to Fung and Keenan (Fung and Keenan, 2014), allowing for a small sample and population size. Observed heterozygosity (*h_o_*) and unbiased expected heterozygosity (*h_e_*) according to Nei (Nei and Chesser, 1983) were estimated using Arlequin v.3.5 software for population genetic analysis (Excoffier and Lischer, 2010). Five randomized datasets of 30 animals were used to calculate *h_o_* to confirm that no sampling size effect would alter diversity estimates. *F_IS_* statistic (the correlation of alleles within an individual relative to the subpopulation) (Weir and Cockerham, 1984) included in Genepop v.4.7.0 was used for estimating potential departures from Hardy-Weinberg equilibrium, using the exact test of significance (Rousset, 2008).

Arlequin v.3.5 was used for calculating the amount of polymorphic sites (S), nucleotide diversity (π), and the average amount of pairwise nucleotide differences between populations (Nei and Li, 1979). MEGAX software (Kumar et al., 2018) was used for calculating the average amount of synonymous (*d*_S_) and nonsynonymous (*d*_N_) substitutions per site by Nei-Gojobori’s method with Jukes-Cantor correction and Z-test was used for assessing *d*_N_/*d*_S_ ratio significance (Nei and Gojobori, 1986).

### Population Structure and Differentiation

Normande population structure was evaluated first because samples from this breed were obtained from three geographically defined regions having local breeding practices which could have differentiated cattle populations. Population structure and genetic differentiation between populations were evaluated by estimating *F_ST_* statistics (the correlation of randomly chosen alleles within the same subpopulation relative to the entire population), as described by Weir and Cockerham (Weir and Cockerham, 1984) using Arlequin v.3.5 (Pairwise *F_ST_*) and Genepop v.4.7.0. (overall *F_ST_*). POPTREE2 (Takezaki et al., 2010) was used for estimating genetic distances *D_A_* (Nei, 1978) from allele frequencies and constructing dendrograms using the NJ algorithm (Takezaki et al., 2010). PAST software v.3.2 (Hammer et al., 2001) was used for assigning confidence levels to branch nodes by bootstrapping *D_A_* genetic distances, with 10,000 replicates. PAST software v.3.2 was used for metric multidimensional scaling (MDS) analysis, based on *D_A_* genetic distances, and principal component analysis (PCA), based on allele frequencies.

### PBR Similarity and Covariation

GeneDoc (Nicholas et al., 1997) was used for calculating identity and similarity percentages (assessed by the BLOSUM62 substitution matrix) for the 31 putative positions constituting the MHC-DRB PBR (Suarez et al., 2006). Percentage similarity was calculated for all observed genotypes within populations; five randomized datasets of 30 individuals from each population were used for one-way ANOVA and Bonferroni tests, after using STATA for proving normal distribution and homoscedasticity (StataCorp, 2017).

WebLogo software (Crooks et al., 2004) was used for creating a logo of PBR positions for each population, using the BLOSUM62 substitution matrix, including a color similarity scheme. Each position’s frequency depended on the allele frequency observed for each population, considering only those having greater than 5% frequency. Covariation was estimated by constructing a vector having 620 coordinates (31 positions × 20 possible aa) for each population. Another Multidimensional Analysis Package (amap R 3.4.3) (R_Core_Team, 2019) was used for calculating Pearson correlation coefficients (PCC) between vectors. The R 3.4.3 stats package (R_Core_Team, 2019) was used to perform a metric MDS on the correlation distance matrix so obtained.

## Results

### *BoLa-DRB3* Allele Distribution Among Normande and Morucha Cattle

Sequence-based typing identified 29 alleles in Spanish Morucha, 23 in Nariño, 27 in Boyacá, and 28 in Cundinamarca Normande cattle (**Table 2**; **Supplementary Data Sheet 1**). No new alleles were observed. Eight (BoLA-DRB3*001:01, 002:01, 005:01, 007:01, 010:01, 012:01, 014:01:01 and 048:02) high-frequency alleles (> 5%) were identified in Normande cattle, accounting for 55.6% of all alleles. Six of these alleles were found to be high-frequency alleles in at least two out of three regions. BoLA-DRB3*001:01 and BoLA-DRB3*002:01 (16.1% cumulative frequency) occurred with high frequency in all three Colombian regions.

**Table 2:** High frequency alleles (>5%) ound fin Cundinamarca Normande, Nariño Normande, Boyacá Normande, overall Normande, and Spanish Morucha.

BoLA-DRB3 allele	Cundinamarca (N = 41)		Nariño (N = 30)		Boyacá (N = 40)		Total Normande (N = 111)		Morucha(N = 54)	
	Observed	CI	Observed	CI	Observed	CI	Observed	CI	Observed	CI
001:01	**0.085**	**0.035 – 0.175**	**0.150**	**0.09 – 0.25**	**0.087**	**0.035 – 0.180**	**0.103**	**0.055 – 0.185**	0.000	0.000 –0.045
002:01	**0.073**	**0.03 – 0.17**	**0.050**	**0.03 – 0.13**	**0.050**	**0.02 – 0.14**	**0.058**	**0.03 – 0.13**	0.000	0.000 – 0.045
003:01	0.000	0.000 – 0.065	0.033	0.02 – 0.12	0.000	0.000 – 0.065	0.009	0.005 – 0.060	**0.120**	**0.070 – 0.195**
005:01	**0.085**	**0.035 – 0.175**	**0.050**	**0.03 – 0.13**	0.025	0.010 – 0.105	**0.054**	**0.025 – 0.125**	**0.111**	**0.065 – 0.190**
007:01	**0.073**	**0.03 – 0.17**	**0.050**	**0.03 – 0.13**	0.025	0.010 – 0.105	**0.049**	**0.025 – 0.125**	0.009	0.005 – 0.050
010:01	0.024	0.010 – 0.105	0.016	0.01 – 0.08	**0.137**	**0.090** – **0.325**	**0.063**	**0.03** – **0.13**	0.028	0.015 – 0.075
012:01	0.024	0.010 – 0.105	0.000	0.00 – 0.07	**0.125**	**0.060** – **0.235**	**0.054**	**0.025** – **0.125**	0.028	0.015 – 0.075
014:01:01	**0.085**	**0.035** – **0.175**	**0.150**	**0.09** – **0.25**	0.012	0.005 – 0.075	**0.076**	**0.035** – **0.150**	0.000	0.000 – 0.045
020:01:01	0.000	0.000 – 0.065	0.000	0.00 – 0.07	0.037	0.015 – 0.115	0.013	0.010 – 0.065	**0.056**	**0.03** – **0.12**
024:06	0.073	0.03 – 0.17	0.000	0.00 – 0.07	0.000	0.000 – 0.065	0.027	0.015 – 0.090	0.000	0.000 – 0.045
048:02	**0.109**	**0.055** – **0.205**	**0.200**	**0.12** – **0.32**	0.012	0.005 – 0.075	**0.099**	**0.05** – **0.18**	**0.231**	**0.15** – **0.30**
	N_a_ = 28		N_a_ = 23		N_a_ = 27		N_a_ = 53		N_a_ = 29	

CI, confidence interval; N_a_, Amount of alleles.

The alleles found to have high frequency for that specific population are shown in boldface.

Regarding Morucha cattle, only four alleles (BoLA-DRB3*003:01, 005:01, 020:01:01 and 048:02) occurred with high frequency, accounting for 51.9% of all alleles. Only BoLA-DRB3*005:01 and BoLA-DRB3*048:02 were shared by both breeds as high-frequency alleles. **Supplementary Table 1** gives complete information about allele frequencies.

### Genetic Variability in the *BoLA-DRB3* Locus and Hardy-Weinberg Equilibrium Regarding Normande and Morucha Breeds

Overall *F_ST_* (*F_ST_* = 0.022) and pairwise *F_ST_* calculated within the Normande breed indicated that the three populations were differentiated at a similar degree to that seen between breeds, and therefore these three Colombian Normande populations were considered separately for diversity analysis.

We determined the observed (*h_o_*) and expected (*h_e_*) heterozygosity and the fixation index (*F_IS_*) in Morucha and Normande cattle populations and compared them to those reported for other cattle breeds to examine *BoLA-DRB3* locus variability and potential departures from Hardy-Weinberg equilibrium. No relation with the original sample size was detected when *h_o_* was calculated from randomized datasets of 30 animals, indicating that this estimate (and its derivatives) can be used to compare genetic diversity in populations having unequal sized samples. For the three Normande populations as well as the Morucha population, *h_e_* was higher than *h_o_* with a significant heterozygote deficiency (**Table 3**). All three Normande populations as well as the Morucha had the lowest *h_o_* compared to the other cattle populations, excluding the Chilean Hereford-Jersey population, although having similar *h_e_* levels. These populations also had the greatest deviations from Hardy-Weinberg equilibrium (*F_IS_*).

**Table 3:** Amount of individuals (N), amount of alleles (N_a_), observed (*h_o_*), and expected (*h_e_*) heterozygosity and Hardy-Weinberg equilibrium, as evaluated by *F_IS_* coefficient, for 21 cattle populations.

Population	N	N_a_	h_o_	h_e_	F_IS_ - *p* value
Cn_Nor	30	23	0.63	0.92	0.3113 – 0.0027
Cc_Nor	41	28	0.68	0.95	0.2848 – < 0.001
Cb_Nor	40	27	0.70	0.94	0.2612 – < 0.001
Sp_Mor	54	29	0.67	0.92	0.2713 – < 0.001
Bo_Ne	116	26	0.78	0.87	0.099 –0.741
Bo_Gir	107	18	0.88	0.92	0.041 – 0.153
Pe_Ne-Br	195	33	0.76	0.86	0.113 – < 0.001
Ar_Ho	424	32	0.84	0.91	0.079 – 0.0040
Bo_Ho	159	23	0.93	0.90	−0.035 – 0.0285
Pa_Ho	127	26	0.83	0.89	0.025 – 0.1036
Pe_Ho	133	20	0.90	0.87	−0.018 – 0.9514
Ch_Ho	113	21	0.84	0.89	0.059 – 0.0006
Ch_He	49	15	0.82	0.87	0.057 – 0.557
Ch_He-Je	65	23	0.69	0.94	0.2639 – < 0.001
Ch_Wa	81	27	0.94	0.92	−0.0208 – 0.7041
Bo_ Yac	113	34	0.92	0.95	0.034 – 0.78
C_HdV	66	23	0.97	0.94	−0.036 – 0.0004
Ja_Ho	101	18	0.92	0.90	−0.022 – 0.3141
Ja_Sh	100	20	0.92	0.91	−0.009 – 0.095
Ja_Je	69	14	0.91	0.89	−0.030 – 0.0017
Ja_Bl	201	23	0.90	0.91	0.009 – 0.362

### Genetic Diversity at Sequence Level and Selection Pattern in *BoLA-DRB3*

The amount of polymorphic sites (S) and nucleotide diversity (π) were estimated to evaluate nucleotide genetic variability within populations (**Table 4**). The four populations had intermediate S values (61 for Spanish Morucha and Cundinamarca Normande to 65 for Nariño Normande and Boyacá Normande), while the π value was the lowest in Spanish Morucha (0.0711) and intermediate in the three Normande populations (0.0790 to 0.0817).

**Table 4:** Values for the amount of polymorphic sites (S), nucleotide diversity (π), mean amount of nonsynonymous (*d_N_*) and synonymous (*d_S_*) nucleotide substitutions per site, in 21 cattle populations.

Population	Nucleotide level		Codon level				Amino acid similarity in PBR	
			β1 domain		PBR			
	S	π	dN	dS	dN	dS	Mean	SD
Cn_Nor	65	0.0790	0.10	0.05*	0.24	0.09*	78.47	7.07
Cc_Nor	61	0.0816	0.10	0.04*	0.23	0.08*	76.85	9.43
Cb_Nor	65	0.0817	0.10	0.05*	0.23	0.09*	75.96	4.53
Sp_Mor	61	0.0711	0.10	0.09	0.22	0.07*	81.61	7.37
Bo_Ne	66	0.0753	0.12	0.05*	0.24	0.07*	78.66	7.04
Bo_Gir	57	0.0829	0.10	0.04*	0.23	0.07*	76.72	7.93
Pe_Ne-Br	67	0.0724	0.12	0.05*	0.23	0.08*	76.9	8.79
Ar_Ho	65	0.0860	0.11	0.08	0.24	0.07*	73.84	8.07
Bo_Ho	65	0.0831	0.11	0.04*	0.25	0.09*	72.77	7.69
Pa_Ho	62	0.0857	0.10	0.09	0.24	0.07*	72.84	8.19
Pe_Ho	63	0.0865	0.11	0.05*	0.24	0.09*	73.5	8.85
Ch_Ho	60	0.0859	0.10	0.04*	0.24	0.08*	72.74	8.8
Ch_He	57	0.0743	0.10	0.03*	0.23	0.07*	74.37	6.85
Ch_He-Je	66	0.0825	0.11	0.04*	0.24	0.08*	75.44	8.16
Ch_Wa	65	0.0798	0.11	0.09	0.23	0.08*	74.91	6.51
Bo_ Yac	67	0.0861	0.11	0.08	0.24	0.07*	73.48	8.32
C_HdV	58	0.0832	0.11	0.03*	0.24	0.07*	NA	NA
Ja_Ho	58	0.0851	0.10	0.04*	0.23	0.08*	74.31	6.44
Ja_Sh	58	0.0841	0.10	0.04*	0.24	0.08*	NA	NA
Ja_Je	57	0.0719	0.11	0.04*	0.23	0.07*	77.97	8.56
Ja_Bl	69	0.0803	0.10	0.05*	0.23	0.09*	NA	NA

Mean and standard deviation (SD) for similarity percentage for all genotypes observed within 18 populations, as evaluated by the BLOSUM 62 substitution matrix. Asterisks indicate a significant d_N_/d_S_ ratio.

**Figure 1:**
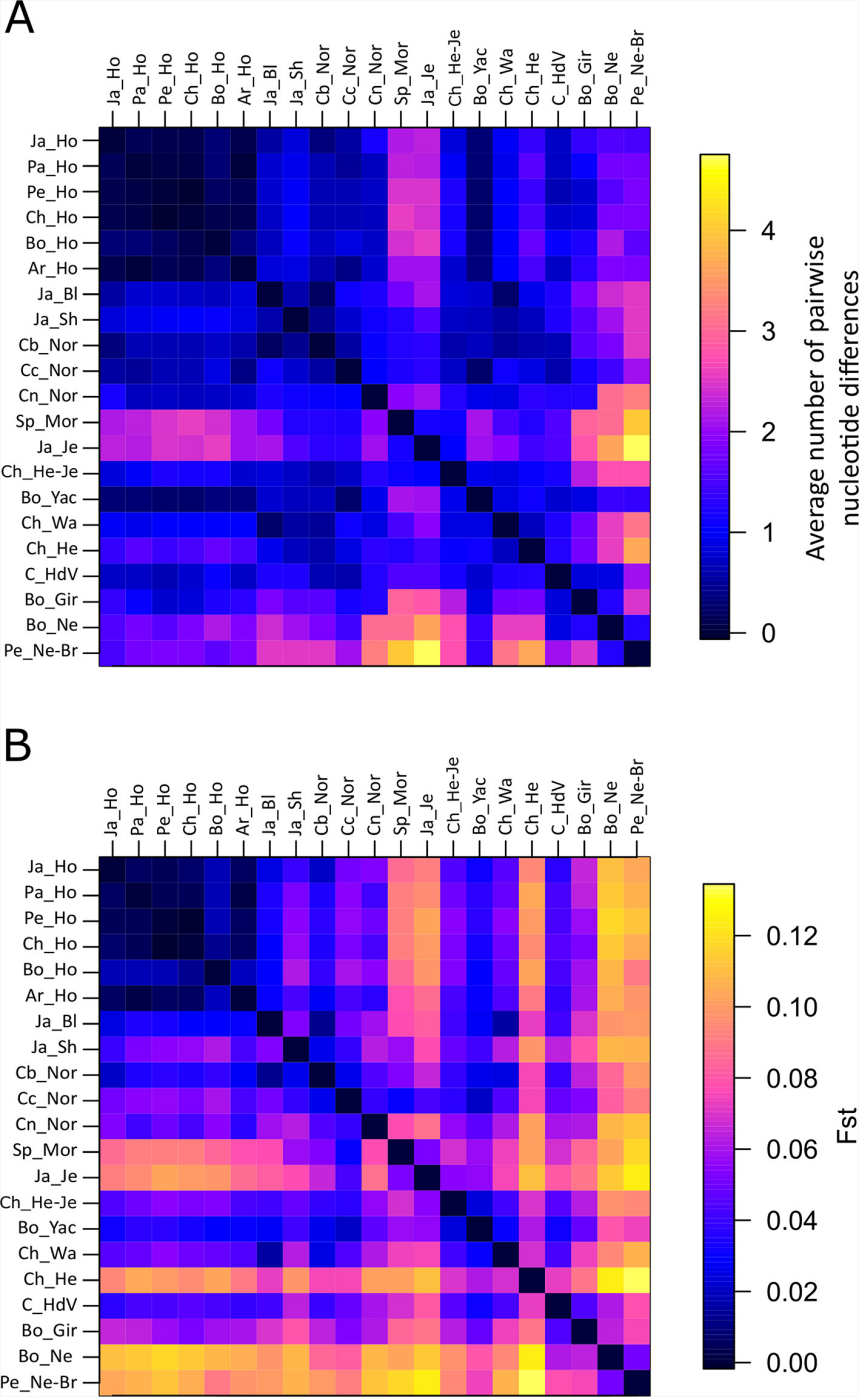
Heat map of the calculated average amount of pairwise nucleotide differences **(A)** and **(B)** pairwise *F_ST_* among 21 cattle populations. Identical values are shown above and below the diagonal.

Nucleotide genetic variability between populations was also assessed calculating the average amount of pairwise nucleotide differences (**Figure 1A**; **Supplementary Data Sheet 2**), which ranged from -0.0367 (between Chilean Holstein and Peruvian Holstein) to 4.73 (between Chilean Hereford-Jersey and Peruvian Nellore-Brahman). Nucleotide differences between populations can be negative in rare cases where nucleotide diversity is high because it implies subtracting variability within populations from total variability (Nei and Li, 1979).

Holstein populations had rather low differentiation from each other in the average amount of pairwise nucleotide differences. Bo_Yac was observed to have little differentiation from Holstein populations. Four comparisons had the highest nucleotide variability values (Peruvian Nellore-Brahman with Japanese Jersey, Peruvian Nellore-Brahman with Spanish Morucha, Bolivian Nellore with Japanese Jersey, and Peruvian Nellore-Brahman with Chilean Hereford). The remaining comparisons had intermediate values. By contrast with Normande populations, Spanish Morucha had strong differentiation from Holstein and zebu populations, similar to that observed in Japanese Jersey. Boyacá Normande was more differentiated from Cundinamarca Normande than from Nariño Normande. Nariño Normande had the highest differentiation from zebu populations.

Codon selection was evaluated using the *d*_N_/*d*_S_ ratio. Selection pattern was very similar for Normande and Spanish Morucha populations, as well as between all populations (**Table 4**). Considering the whole β1 domain, the estimated nonsynonymous substitution rate (*d*_N_) was 0.10, in both Spanish Morucha and Normande cattle and the synonymous substitutions rate (*d*_S_) ranged from 0.04 in Cundinamarca Normande to 0.09 in Spanish Morucha, with a significant *d*_N_/*d*_S_ ratio being found in most populations. The difference between *d*_N_ and *d*_S_ was more prominent when only considering PBR codons and all populations had a significant *d*_N_/*d*_S_ ratio. Spanish Morucha had the lowest *d*_N_ value of all populations (0.22) while sharing the lowest *d*_S_ value (0.07) with other populations.

### Population Structure and Genetic Differentiation

Pairwise *F_ST_* values were used for assessing genetic differentiation between populations (**Figure 1B**; **Supplementary Data Sheet 3**). Pairwise comparisons ranged from −0.0010 (Chilean Holstein with Peruvian Holstein) to 0.1338 (Chilean Hereford with Peruvian Nellore-Brahman). Similar to that seen regarding the amount of pairwise nucleotide differences, the lowest *F_ST_* values were observed when comparing Holstein populations to one another. Nevertheless, except for Bolivian Gir, zebu populations had marked differentiation compared to taurine populations. Similar to that seen in Chilean Hereford and Japanese Jersey, Spanish Morucha was differentiated more from other taurine populations than Normande (0.0688 average pairwise *F_ST_* in Spanish Morucha and 0.0470 in Normande). Nariño Normande was also observed to be most divergent from zebu populations and Boyacá Normande was less differentiated from taurine populations than Boyacá Normande and Nariño Normande. The highest differentiation was observed between Bolivian Nellore with Holstein populations and when comparing a group consisting of Japanese Jersey, Spanish Morucha, and Chilean Hereford to Bolivian Nellore and Peruvian Nellore-Brahman. The remaining comparisons had an intermediate level of differentiation.

**Figure 2:**
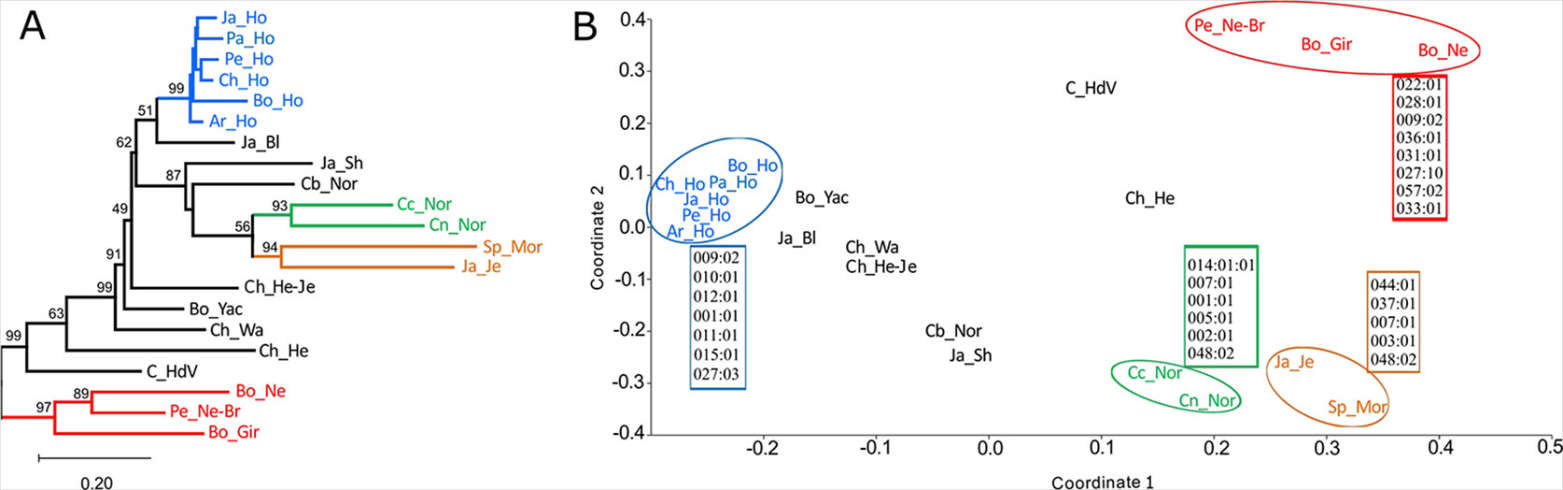
Dendrogram **(A)** and multidimensional scaling (MDS) analysis **(B)** based on *D_A_* genetic distances. Alleles are indicated which contributed to population grouping, as identified by principal component analysis (PCA). Groups well-supported by bootstrap in MDS are circled in the dendrogram.

The *D_A_* genetic distance matrix was used to construct a dendrogram, using the NJ algorithm (**Figure 2A** and **Supplementary Data Sheet 4**). *D_A_* genetic distances were also used for depicting the relationship between populations in two dimensions by MDS analysis (**Figure 2B**). Dendrograms and MDS gave a similar distribution pattern, only differing regarding the clustering of groups not well supported by bootstrap (Japanese Black, Japanese Shorthorn, Boyacá Normande, Chilean Hereford-Jersey, Bo_Yac, Chilean Wagyu, Colombian Hartón del Valle and Chilean Hereford). MDS analysis had a 0.799 R-square (RSQ), indicating that representation in two coordinates gave a good description of real *BoLA-DRB3* variability. Zebu populations were clearly separated from taurine populations. Despite the two types of cattle having a heterogeneous distribution, lacking any clear clustering except for the Holstein group, we were able to identify four clusters which were relatively well supported by bootstrap (>90). The zebu group (Bolivian Nellore, Peruvian Nellore-Brahman and Bolivian Gir), the Holstein group (Japanese Holstein, Peruvian Holstein, Peruvian Holstein, Chilean Holstein, Bolivian Holstein and Argentinian Holstein), a group formed by Cundinamarca Normande and Nariño Normande and a group formed by Spanish Morucha and Japanese Jersey. Boyacá Normande was noted to be closer to the other taurine populations than Cundinamarca Normande and Nariño Normande.

Allele frequencies were used for PCA and these results were compared to MDS to identify which alleles contributed most to differentiate or cluster cattle populations. Groups clearly identified in both MDS and in the first two PC were depicted as having the major alleles contributing to such grouping (**Figure 2B** and **Supplementary Data Sheet 5**).

Population distribution in the first two principal components (PC) (**Supplementary Figure 1**), accounting for 44.6% of total variance, was very similar to MDS. Boyacá Normande was located toward the Holstein variability region in the first PC and was clearly differentiated from Spanish Morucha, and the other Normande, which were located toward zebu populations’ variability region. Boyacá Normande differentiation in the first PC was explained by the high BoLA-DRB3*010:01, 012:01, 001:01, and 011:01 allele frequency. The different position of Cundinamarca Normande and Nariño Normande regarding Spanish Morucha in the first PC was explained by BoLA-DRB3*007:01, 002:01, 014:01:01, and 001:01 alleles. Holstein populations were clearly differentiated in the first PC by high BoLA-DRB3*009:02, 010:01, 012:01, 001:01, 011:01, and 015:01 allele frequency. Alleles BoLA-DRB3*048:02, 002:01, 003:01, 005:01, 007:01, and 008:01 explained the grouping of Cundinamarca Normande, Nariño Normande, Spanish Morucha, and Japanese Jersey in the second PC. Moreover, BoLA-DRB3*012:01, 016:01, 010:01, and 011:01 alleles differentiated Boyacá Normande, while BoLA-DRB3*048:02, 001:01, 014:01:01, and 024:06 alleles differentiated Cundinamarca Normande from Nariño Normande.

The clearest differentiation between zebu and taurine populations was observed in the second PC and was mainly explained by BoLA-DRB3*036:01, 028:01, 009:02, 031:01, 027:10, 057:02, 033:01, and 022:01 alleles, enriched in zebu populations. Spanish Morucha clustered with Japanese Jersey in the first two PC, enriched with BoLA-DRB3*048:02, 044:01, 037:01, 007:01, and 003:01. Nariño Normande and Cundinamarca Normande were grouped by BoLA-DRB3*014:01:01, 007:01, 001:01, 005:01, 002:01, and 048:02 alleles. Several alleles occurring with low frequency, unique to some populations (low correlation in the PCA), contributed to differentiating these populations (BoLA-DRB3*020:09, 024:02, 024:04, 012:02, 020:10, 024:01, 030:22, and 078:01 in Spanish Morucha, BoLA-DRB3*043:02, 022:04, 022:05, 024:07, 028:03, 029:01, and 051:01 in Nariño Normande, BoLA-DRB3*019:02, 034:03, and BoLA-DRB3*075:03 in Cundinamarca Normande) (**Supplementary Data Sheet 5**).

### PBR Sequence Similarity and Covariation Between Populations

PBR position similarity was calculated for the alleles reported in the populations used in this study and from previous reports (Takeshima et al., 2003; Giovambattista et al., 2013; Takeshima et al., 2015a; Takeshima et al., 2015b; Takeshima et al., 2018) to further analyze *BoLA-DRB3* diversity (**Table 4**, **Supplementary Data Sheet 6**). Normande cattle had 78.47% similarity for Nariño, 76.85% for Cundinamarca, and 75.96% for Boyacá. Spanish Morucha similarity was 81.61%, this being the highest value for all groups evaluated here. Holstein populations had an average 74% similarity, Bolivian Holstein (72.77%), and Paraguayan Holstein (72.84%) having the lowest PBR similarity. Average similarity for zebu populations was 76.81% (76.90% for Peruvian Nellore-Brahman, 76.72% for Bolivian Gir, and 78.66% for Bolivian Nellore).

Five randomized sets of 30 animals were constructed to reduce possible sampling effects, considering the clustering observed in diversity analysis results. The groups compared were Holsteins, zebu populations, Nariño Normande, Cundinamarca Normande, Boyacá Normande, and Spanish Morucha. Significant differences between the evaluated groups were found for all randomized datasets (≤0.05 *p*-value). The Bonferroni test gave significant differences between Spanish Morucha and Holsteins populations (≤0.05 *p*-value) for all randomizations. Holstein populations’ percentage similarity was lower than for Spanish Morucha (81.61%), indicating that Spanish Morucha had less diversity in the PBR. When identity in PBR positions was assessed, we found that 129 out of 140 alleles had a different PBR.

**Figure 3:**
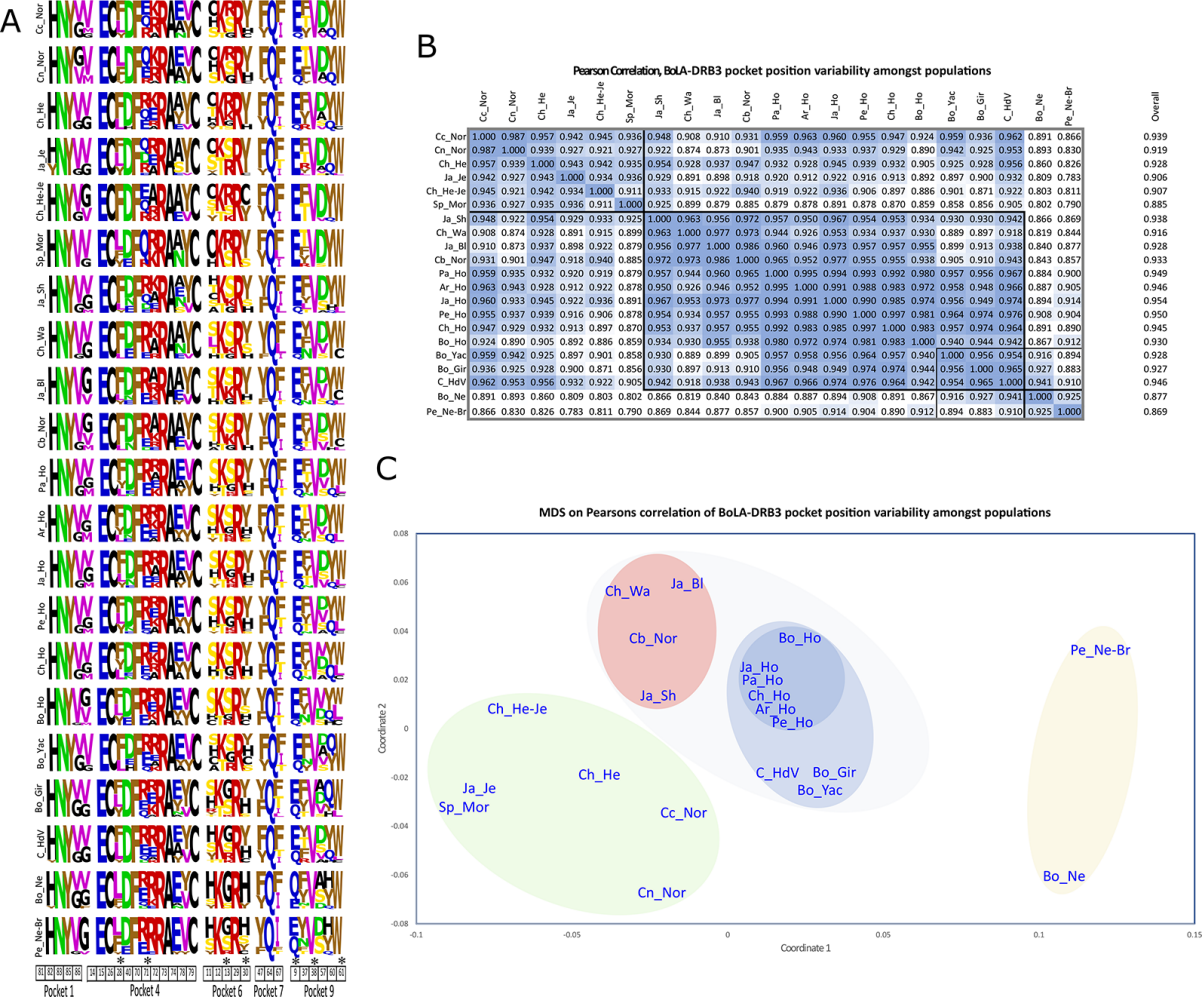
Peptide-binding region (PBR) logos **(A)**, Pearson correlation coefficient **(B)** and multidimensional scaling (MDS) matrix **(C)** based on *BoLA-DRB3* pocket position variability among populations. In A, similar colors indicate conservative and different colors nonconservative aa changes according to the BLOSUM 62 substitution matrix. B shows identical values above and below the diagonal. * Positions shared by more than one pocket.

The logo representation of PBR had remarkable similarities among populations, having very similar substitution patterns (**Figure 3A**). PBR logos had highly variable positions (11, 13, 37, 70, 71, and 74), some of which were shared by several pockets and usually had nonconservative substitutions. Covariation analysis of PBR aa sequence logos for all cattle populations (**Figure 3B**) showed that most variability was due to differences in aa frequencies and not aa variations in the same position.

Global covariation between PBR positions was high (PCC = 0.92). Three groups could be discriminated (**Figures 3B**, **C**). The first contained Cundinamarca Normande, Nariño Normande, Chilean Hereford, Japanese Jersey, Chilean Hereford-Jersey, and Spanish Morucha (group PCC = 0.94). The second included Japanese Shorthorn, Chilean Wagyu, Japanese Black, Boyacá Normande, Paraguayan Holstein, Argentinian Holstein, Japanese Holstein, Peruvian Holstein, Chilean Holstein, Bolivian Holstein, Bo_Yac, Bolivian Gir, and Colombian Hartón del Valle (group PCC = 0.96) and the third consisted of Peruvian Nellore-Brahman and Bolivian Nellore (group PCC = 0.93). All taurine populations and zebu Bo-Gir had an average 0.94 PCC. Although these groups were different regarding previous analysis, similar groups were found, distinguishing zebu populations (Peruvian Nellore-Brahman – Bolivian Nellore), Holstein populations and the Spanish Morucha - Japanese Jersey and Cundinamarca Normande – Nariño Normande association. Overall covariation gave Spanish Morucha as the most differentiated population between taurine breeds, similar to zebu Bolivian Nellore and Peruvian Nellore-Brahman.

## Discussion

MHC gene analysis can provide information about domestic species’ level of overall genetic diversity. While several studies have shown decreased MHC variability in association with population bottlenecks (Bollmer et al., 2007; Bollmer et al., 2011; Mason et al., 2011; Zhang et al., 2016), others have found that high variability persists despite extreme population bottlenecks (Edwards and Hedrick, 1998; Garrigan and Hedrick, 2001; Aguilar et al., 2004; Borg et al., 2011; Moutou et al., 2013; Newhouse and Balakrishnan, 2015). The *BoLA-DRB3* gene is the most polymorphic bovine MHC class II DR gene and is the only one having been shown to be fully functional, although the total amount of alleles is low compared to species such as humans (Shiina et al., 2017). This study was therefore aimed at describing *BoLA-DRB3* genetic diversity in two new cattle breeds and analyzed whether domestic cattle breeding has decreased actual MHC variability.

Several measures of genetic diversity, such as *h_s_* or *F_IS_*, have corrections for sample size (Nei and Chesser, 1983). As expected, *h_o_* estimated for random datasets did not depend on original sample size. This was because *h_o_* is an unbiased estimator of parametric value (Nei and Chesser, 1983) and is mainly determined by the sampling method used. Therefore, the lower *h_o_* and higher *F_IS_* value for Spanish Morucha and Colombian Normande compared to the other cattle populations (Takeshima et al., 2003; Giovambattista et al., 2013; Takeshima et al., 2015a; Takeshima et al., 2015b; Takeshima et al., 2018) indicated heterozygote deficiency for these two breeds.

Drift and selection interact to configure the allele distribution observed in populations. MHC (a nonneutral marker) allele distribution would have been expected to be that of balancing selection with excess heterozygotes (Hughes and Nei, 1988; Hughes and Yeager, 1998; Takeshima et al., 2008). Nevertheless, the heterozygote deficiency observed in Spanish Morucha and Colombian Normande suggested that other evolutionary processes, such as inbreeding and/or bottlenecks, would have been acting on these populations. Genetic improvement regarding dairy (Normande) or beef production (Morucha) traits or bottlenecks in these breeds’ origin might have contributed to the low diversity observed and high level of inbreeding inferred (>0.2 in Normande and Morucha) for the *BoLA-DRB3* locus. Decreased MHC diversity can result in a narrower spectrum of pathogens which a population can recognize, less viability and even loss of pregnancies due to maternal-foetal interactions (Sommer, 2005). However, functional diversity could be preserved despite few alleles being involved.

*BoLA-DRB3* divergence between Colombian Normande populations was even higher in some cases than the divergence observed between breeds on different continents (e.g., Spanish Morucha and Japanese Jersey). *BoLA-DRB3* divergence between Normande populations can be highlighted compared to that for Holstein populations which are not so differentiated, despite also being phenotypically homogeneous. Comparing French and Colombian Normande cattle might provide insights into Normande populations’ regional divergence because the French Normande breed is slightly variable and is considered a completely closed breed (Danchin-Burge et al., 2012; Porter et al., 2016).

Spanish Morucha is an autochthonous cattle breed from the black Iberian group which is restricted to the Northwest of the Iberian Peninsula; it was initially used for draft, milk and beef but is now solely used for meat production. It has moderate inbreeding rates and a population structure restricting mating within herds (Canas-Alvarez et al., 2014). Nevertheless, a restricted gene flow regarding other autochthonous Spanish breeds having geographical proximity during their history and similar production systems might have moulded Spanish Morucha genetic diversity (Canas-Alvarez et al., 2015). As such, this breed has had relatively low human intervention and can thus be considered close to a natural cattle population.

Spanish Morucha has been shown to be more differentiated from other taurine and zebu breeds. Likewise, Japanese Jersey and Chilean Hereford pairwise *F_ST_* values for Spanish Morucha were the highest among taurine populations. A similar trend was found when analyzing the sequence information for each allele through the average amount of pairwise nucleotide differences, Spanish Morucha and Japanese Jersey being more differentiated. BoLA-DRB3*048:02 high frequency was the main reason for Spanish Morucha and Japanese Jersey clustering. As European cattle were introduced to improve Japanese native breeds, a similar *BoLA-DRB3* gene pool in Iberian and Jersey founder populations or the direct introduction of Iberian cattle along with similar selective pressures might have led to these two cattle populations’ characteristic divergence, despite these two breeds having different production features and purposes.

The frequency of an MHC allele or group of alleles might become increased in cattle populations having the same selective pressures imposed by similar production systems, even in different geographic locations. From the analysis of previously published data (Takeshima et al., 2015a), we found that Holstein populations were enriched with alleles reported to be associated with resistance (BoLA-DRB3*011:01 and BoLA-DRB3*012:01) and susceptibility (BoLA-DRB3*001:01 and BoLA-DRB3*015:01) to mastitis (Yoshida et al., 2009b; Yoshida et al., 2009a). The Holstein breed has been submitted to intense selective pressure due to milk production traits which can make it susceptible to mastitis but, at the same time, there is also pressure for selecting resistant individuals. These two forces might thus create high frequencies of alleles associated with susceptibility and resistance to be in balance. However, it is worth noting that Japanese Black (a beef breed) was also enriched by these alleles. No particular enrichment with this kind of alleles was observed for Normande or Morucha cattle.

The greatest variability in the β1 domain is located in PBR positions, thereby determining the peptides to which an allele can bind. In agreement with other diversity analysis in which Spanish Morucha had lower nucleotide and codon variability, variability in the PBR was lower and could indicate that the magnitude of this population’s peptide repertoire could be significantly smaller than that of other cattle. Holstein populations shared similar PBR constitution and covariation patterns by contrast with Normande ones, which had a more differentiated PBR. Despite zebu populations being well-differentiated, Bolivian Gir appears to have a *BoLA-DRB3* allele distribution similar to that of taurine breeds, thus grouping them with taurine populations. High correlation coefficients in PBR aa sequence logo analysis indicated that all breeds tended to have similar peptide repertoires, despite genetic differentiation. This suggested that domestication and/or breeding have not decreased functional MHC variability. Indeed, the fact that Spanish Morucha has lesser variability suggests that breeding might have increased both genetic and functional variation.

Great diversity was observed when analysing *BoLA-DRB3* identity in the PBR, despite having fewer alleles compared to other species, such as primates (Suarez et al., 2006), i.e., even though having fewer alleles, potential peptide repertoire size in bovines is similar to that for other species. This means that cattle have 129 distinct PBR (based on sequence identity) and that infectious disease control programmes should be designed as if there were 129 different alleles instead of 140 (136 in the IPD Database and four reported elsewhere). A similar amount of PBR sequences has been determined in humans and owl monkeys (Suarez et al., 2017). This is especially important when it comes to peptide-based vaccine design which might be more efficacious when the peptide-MHC complex has potentially greater affinity as this can be increased by modifying peptides to bind to MHC molecules [reviewed in (Patarroyo et al., 2011)].

However, allele frequency would be the main guide regarding population control programmes based on MHC diversity. For example, based on allele frequency distribution, 67% to 73% of the peptide repertoire in Holstein populations would be determined only by seven alleles and this information could be used when designing peptide-based vaccines. A potential limitation of such approach is that the MHC-DR PBR positions used have been documented by X-ray crystallography just in humans and mice. Nevertheless, several of these positions have been associated with vaccine responses and susceptibility or resistance to infectious diseases (Garcia-Briones et al., 2000; Baxter et al., 2009; Rastislav and Mangesh, 2012; Carignano et al., 2017) and thus analysis based on these positions is reasonable.

## Conclusions

Despite genetic divergence being observed between cattle populations, their peptide repertoires seem to be functionally equivalent. The great similarities and covariation among populations regarding the *BoLA-DRB3* PBR might signify that *BoLA-DRB3* peptide repertoire size is very similar among the cattle populations analyzed here and thus breeding has not reduced cattle’s MHC functional diversity. Although cattle have fewer alleles than species such as some primates, PBR sequence identity supports the idea that the potential peptide repertoire between these species is equivalent and functional diversity can persist despite population bottlenecks. In other words, less genetic MHC diversity (fewer alleles) does not mean loss of functional diversity. Genetic improvement regarding breeding and domestication appears to have produced three different Colombian Normande populations based on *BoLA-DRB3* genetic diversity. When analysing these populations regarding the diversity described for other cattle, it was noted that the forces which have contributed toward moulding *BoLA-DRB3* locus diversity in Colombian Normande have produced remarkable differentiation compared to other phenotypically homogeneous breeds, such as Holstein. It is also remarkable that Spanish Morucha was shown to be strongly differentiated in all types of analysis.

## Data Availability Statement

All datasets generated for this study are included in the article/**Supplementary Material**.

## Ethics Statement

The animal study was reviewed and approved by The Universidad de Ciencias Aplicadas y Ambientales (UDCA, Bogotá) Research Ethics Committee (Minute No. 201901) approved this study.

## Author Contributions

MB, DO, CS, and MP designed the study. MB, DO, and CV carried out experimental procedures. MB, DO, CS, BV, CV, JL-A, AM, IO, and MP analyzed the data and wrote the manuscript. AM and MP supervised the study. All authors approved the final version of the manuscript.

## Funding

This study was partially supported by the Diputación de Salamanca, Caja Rural de Salamanca and the Instituto de Salud Carlos III, ISCIII, Spain (www.isciii.es) under grants: RICET RD16/0027/0018 and PI16/01784. MB was partially financed trough a scholarship from the Microbiology Postgraduate Program, Universidad Nacional de Colombia. DO was financed through a scholarship from the PhD Program in Biomedical and Biological Sciences, Universidad del Rosario.

## Conflict of Interest

The authors declare that the research was conducted in the absence of any commercial or financial relationships that could be construed as a potential conflict of interest.

## Abbreviations

ANOVA, analysis of variance; BoLA, bovine leukocyte antigen; MDS, multidimensional scaling analysis; MHC, major histocompatibility complex; PBR, peptide-binding region; aa, amino acid; PCA, principal component analysis; PCC, Pearson correlation coefficient.
