# Supplementary material for: Major Histocompatibility Complex Class II (DRB3) Genetic Diversity in Spanish Morucha and Colombian Normande Cattle Compared to Taurine and Zebu Populations
Source: Front Genet. 2020 Jan 10;10:1293. doi: 10.3389/fgene.2019.01293 (PMC6965167; doi:10.3389/fgene.2019.01293)
Supplement: Supplementary file 7 [file Image_1.pdf]

Supplementary Material

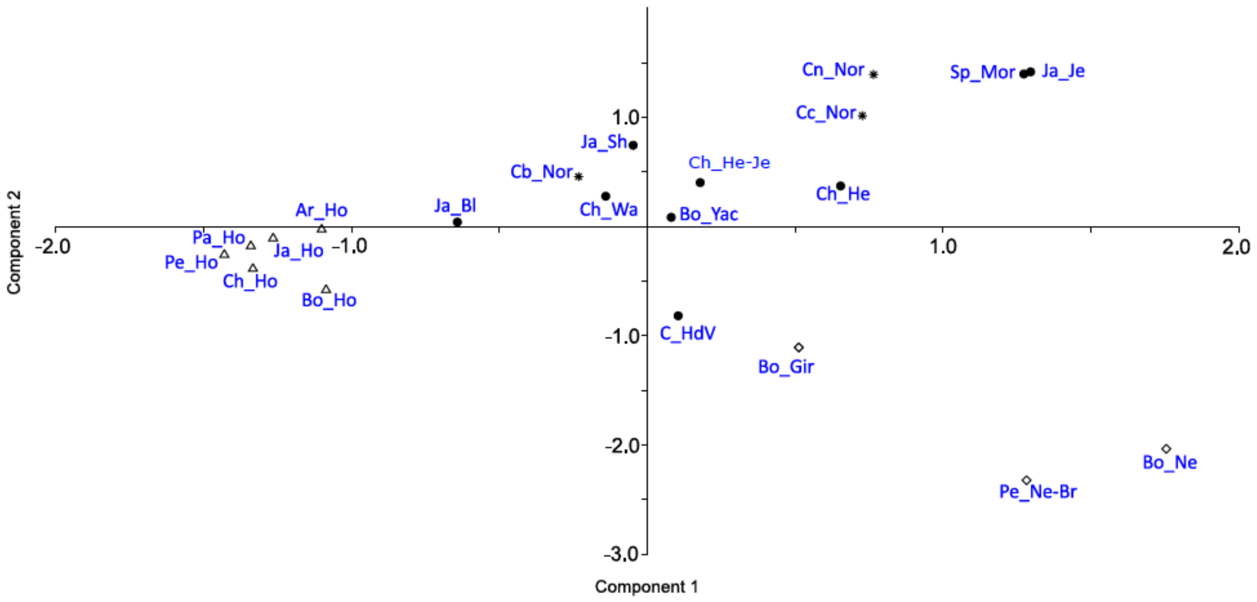

Supplementary Figure 1. Plot of the two principal components based on allele frequency for 21 cattle populations.
