# Supplementary material for: Major Histocompatibility Complex Class II (DRB3) Genetic Diversity in Spanish Morucha and Colombian Normande Cattle Compared to Taurine and Zebu Populations
Source: Front Genet. 2020 Jan 10;10:1293. doi: 10.3389/fgene.2019.01293 (PMC6965167; doi:10.3389/fgene.2019.01293)
Supplement: Supplementary file 8 [file Table_1.pdf]

Supplementary Table 1. Allele frequencies and confidence intervals observed for Colombian (Nariño, Cundinamarca, Boyacá and total) Normande and Sp_Mor cattle.

BoLA-DRB3 Allele	Cundinamarca N = 41		Boyacá N = 40		Nariño N = 30		Total Normande N = 111		Morucha N = 54	
	Observed	IC	Observed	IC	Observed	IC	Observed	IC	Observed	IC
001:01	**0,085**	**0.035 - 0.175**	**0,087**	**0.035 - 0.180**	**0,15**	**0.09 - 0.25**	**0,103**	**0.055 - 0.185**	0	0.000 - 0.045
002:01	**0,073**	**0.03 - 0.17**	**0,05**	**0.02 - 0.14**	**0,05**	**0.03 - 0.13**	**0,058**	**0.03 - 0.13**	0	0.000 - 0.045
003:01	0	0.000 - 0.065	0	0.000 - 0.065	0,033	0.02 - 0.12	0,009	0.005 - 0.060	**0,120**	**0.070 - 0.195**
003:02:01	0	0.000 - 0.065	0	0.000 - 0.065	0	0.00 - 0.07	0	0.000 - 0.055	0,019	0.01 - 0.07
005:01	**0,085**	**0.035 - 0.175**	0,025	0.010 - 0.105	**0,05**	**0.03 - 0.13**	**0,054**	**0.025 - 0.125**	**0,111**	**0.065 - 0.190**
005:02	0	0.000 - 0.065	0	0.000 - 0.065	0,033	0.02 - 0.12	0,009	0.005 - 0.055	0	0.000 - 0.045
005:03	0	0.000 - 0.065	0,012	0.005 - 0.075	0	0.00 - 0.07	0,004	0.005 - 0.060	0	0.000 - 0.045
006:01	0	0.000 - 0.065	0,037	0.015 - 0.115	0	0.00 - 0.07	0,013	0.010 - 0.065	0,009	0.005 - 0.050
007:01	**0,073**	**0.03 - 0.17**	0,025	0.010 - 0.105	**0,05**	**0.03 - 0.13**	**0,049**	**0.025 - 0.125**	0,009	0.005 - 0.050
007:02	0,036	0.015 - 0.110	0	0.000 - 0.065	0	0.00 - 0.07	0,013	0.010 - 0.065	0	0.000 - 0.045
008:01	0,024	0.010 - 0.105	0,05	0.02 - 0.14	0,016	0.01 - 0.08	0,031	0.015 - 0.090	0	0.000 - 0.045
009:01	0	0.000 - 0.065	0,012	0.005 - 0.075	0	0.00 - 0.07	0,004	0.005 - 0.060	0	0.000 - 0.045
010:01	0,024	0.010 - 0.105	**0,137**	**0.090 - 0.325**	0,016	0.01 - 0.08	**0,063**	**0.03 - 0.13**	0,028	0.015 - 0.075
010:02	0,012	0.005 - 0.070	0	0.000 - 0.065	0	0.00 - 0.07	0,004	0.005 - 0.060	0,028	0.015 - 0.075
011:01	0	0.000 - 0.065	0,087	0.035 - 0.180	0	0.00 - 0.07	0,031	0.015 - 0.090	0,009	0.005 - 0.050
011:02	0,036	0.015 - 0.110	0	0.000 - 0.065	0,016	0.01 - 0.08	0,018	0.015 - 0.090	0	0.000 - 0.045
012:01	0,024	0.010 - 0.105	**0,125**	**0.060 - 0.235**	0	0.00 - 0.07	**0,054**	**0.025 - 0.125**	0,028	0.015 - 0.075
012:02	0	0.000 - 0.065	0	0.000 - 0.065	0	0.00 - 0.07	0	0.000 - 0.055	0,009	0.005 - 0.050
013:01	0	0.000 - 0.065	0,025	0.010 - 0.105	0	0.00 - 0.07	0,009	0.005 - 0.055	0	0.000 - 0.045

014:01:01	**0,085**	**0.035 - 0.175**	0,012	0.005 - 0.075	**0,15**	**0.09 - 0.25**	**0,076**	**0.035 - 0.150**	0	0.000 - 0.045
015:05	0,024	0.010 - 0.105	0,012	0.005 - 0.075	0,016	0.01 - 0.08	0,018	0.010 - 0.065	0	0.000 - 0.045
016:01	0	0.000 - 0.065	0,05	0.02 - 0.14	0	0.00 - 0.07	0,018	0.010 - 0.065	0	0.000 - 0.045
017:01	0	0.000 - 0.065	0	0.000 - 0.065	0,033	0.02 - 0.12	0,009	0.005 - 0.055	0	0.000 - 0.045
018:01	0,036	0.015 - 0.110	0	0.000 - 0.065	0	0.00 - 0.07	0,013	0.010 - 0.065	0,019	0.01 - 0.07
019:02	0,012	0.005 - 0.070	0	0.000 - 0.065	0	0.00 - 0.07	0,004	0.005 - 0.060	0	0.000 - 0.045
020:01:01	0	0.000 - 0.065	0,037	0.015 - 0.115	0	0.00 - 0.07	0,013	0.010 - 0.065	**0,056**	**0.03 - 0.12**
020:01:02	0	0.000 - 0.065	0,037	0.015 - 0.115	0,016	0.01 - 0.08	0,018	0.010 - 0.065	0,037	0.02 - 0.10
020:02	0,024	0.010 - 0.105	0	0.000 - 0.065	0	0.00 - 0.07	0,009	0.005 - 0.055	0	0.000 - 0.045
020:03	0,012	0.005 - 0.070	0	0.000 - 0.065	0	0.00 - 0.07	0,004	0.005 - 0.060	0	0.000 - 0.045
020:09	0	0.000 - 0.065	0	0.000 - 0.065	0	0.00 - 0.07	0	0.000 - 0.055	0,019	0.01 - 0.07
020:10	0	0.000 - 0.065	0	0.000 - 0.065	0	0.00 - 0.07	0	0.000 - 0.055	0,009	0.005 - 0.050
021:01	0,012	0.005 - 0.070	0,025	0.010 - 0.105	0	0.00 - 0.07	0	0.000 - 0.055	0	0.000 - 0.045
022:01	0	0.000 - 0.065	0	0.000 - 0.065	0	0.00 - 0.07	0,013	0.010 - 0.065	0	0.000 - 0.045
022:04	0	0.000 - 0.065	0	0.000 - 0.065	0,016	0.01 - 0.08	0,004	0.005 - 0.060	0	0.000 - 0.045
022:05	0	0.000 - 0.065	0	0.000 - 0.065	0,016	0.01 - 0.08	0,004	0.005 - 0.060	0	0.000 - 0.045
023:01	0	0.000 - 0.065	0,012	0.005 - 0.075	0	0.00 - 0.07	0,004	0.005 - 0.060	0,046	0.025 - 0.100
024:01	0	0.000 - 0.065	0	0.000 - 0.065	0	0.00 - 0.07	0	0.000 - 0.055	0,009	0.005 - 0.050
024:02	0	0.000 - 0.065	0	0.000 - 0.065	0	0.00 - 0.07	0	0.000 - 0.055	0,019	0.01 - 0.07
024:04	0	0.000 - 0.065	0	0.000 - 0.065	0	0.00 - 0.07	0	0.000 - 0.055	0,019	0.01 - 0.07
024:06	0,073	0.03 - 0.17	0	0.000 - 0.065	0	0.00 - 0.07	0,027	0.015 - 0.090	0	0.000 - 0.045
024:07	0	0.000 - 0.065	0	0.000 - 0.065	0,016	0.01 - 0.08	0,004	0.005 - 0.060	0	0.000 - 0.045
025:01:01	0,012	0.005 - 0.070	0	0.000 - 0.065	0	0.00 - 0.07	0,004	0.005 - 0.060	0	0.000 - 0.045
026:01	0	0.000 - 0.065	0,025	0.010 - 0.105	0,016	0.01 - 0.08	0,013	0.010 - 0.065	0,009	0.005 - 0.050

027:01	0	0.000 - 0.065	0,012	0.005 - 0.075	0	0.00 - 0.07	0,004	0.005 - 0.060	0	0.000 - 0.045
027:03	0,012	0.005 - 0.070	0	0.000 - 0.065	0	0.00 - 0.07	0,004	0.005 - 0.060	0	0.000 - 0.045
027:07	0,012	0.005 - 0.070	0	0.000 - 0.065	0	0.00 - 0.07	0,004	0.005 - 0.060	0	0.000 - 0.045
028:01	0,012	0.005 - 0.070	0,012	0.005 - 0.075	0	0.00 - 0.07	0,009	0.005 - 0.060	0	0.000 - 0.045
028:02	0,012	0.005 - 0.070	0	0.000 - 0.065	0	0.00 - 0.07	0,004	0.005 - 0.060	0	0.000 - 0.045
028:03	0	0.000 - 0.065	0	0.000 - 0.065	0,016	0.01 - 0.08	0,004	0.005 - 0.060	0	0.000 - 0.045
029:01	0	0.000 - 0.065	0	0.000 - 0.065	0,016	0.01 - 0.08	0,004	0.005 - 0.060	0	0.000 - 0.045
030:22	0	0.000 - 0.065	0	0.000 - 0.065	0	0.00 - 0.07	0	0.000 - 0.055	0,009	0.005 - 0.050
031:01	0	0.000 - 0.065	0,025	0.010 - 0.105	0	0.00 - 0.07	0,009	0.005 - 0.055	0	0.000 - 0.045
031:04	0	0.000 - 0.065	0,012	0.005 - 0.075	0	0.00 - 0.07	0,004	0.005 - 0.060	0	0.000 - 0.045
032:01	0,036	0.015 - 0.110	0	0.000 - 0.065	0	0.00 - 0.07	0,013	0.010 - 0.065	0,019	0.01 - 0.07
032:02	0	0.000 - 0.065	0	0.000 - 0.065	0	0.00 - 0.07	0	0.000 - 0.055	0,009	0.005 - 0.050
034:03	0,012	0.005 - 0.070	0	0.000 - 0.065	0	0.00 - 0.07	0,004	0.005 - 0.060	0	0.000 - 0.045
037:01	0	0.000 - 0.065	0	0.000 - 0.065	0	0.00 - 0.07	0	0.000 - 0.055	0,019	0.01 - 0.07
040:01	0	0.000 - 0.065	0	0.000 - 0.065	0,016	0.01 - 0.08	0,004	0.005 - 0.060	0	0.000 - 0.045
043:01	0	0.000 - 0.065	0	0.000 - 0.065	0	0.00 - 0.07	0	0.000 - 0.055	0,019	0.01 - 0.07
043:02	0	0.000 - 0.065	0	0.000 - 0.065	0,033	0.02 - 0.12	0,009	0.005 - 0.055	0	0.000 - 0.045
044:01	0	0.000 - 0.065	0,012	0.005 - 0.075	0	0.00 - 0.07	0,004	0.005 - 0.060	0,046	0.025 - 0.100
048:02	**0,109**	**0.055 - 0.205**	0,012	0.005 - 0.075	**0,2**	**0.12 - 0.32**	**0,099**	**0.05 - 0.18**	**0,231**	**0.15 - 0.30**
051:01	0	0.000 - 0.065	0	0.000 - 0.065	0,016	0.01 - 0.08	0,004	0.005 - 0.060	0	0.000 - 0.045
058:01	0	0.000 - 0.065	0	0.000 - 0.065	0	0.00 - 0.07	0	0.000 - 0.055	0,028	0.015 - 0.075
070:01	0,012	0.005 - 0.070	0,025	0.010 - 0.105	0	0.00 - 0.07	0,013	0.010 - 0.065	0	0.000 - 0.045
075:03	0,012	0.005 - 0.070	0	0.000 - 0.065	0	0.00 - 0.07	0,004	0.005 - 0.060	0	0.000 - 0.045
078:01	0	0.000 -	0	0.000 -	0	0.00 -	0	0.000 -	0,009	0.005 -

		0.065		0.065		0.07		0.055		0.050
	N_a = 28		N_a = 27		N_a = 23		N_a = 53		N_a = 29	
